# Pneumothorax in connective tissue disease-associated interstitial lung disease

**DOI:** 10.1371/journal.pone.0235624

**Published:** 2020-07-07

**Authors:** Koji Nishimoto, Tomoyuki Fujisawa, Katsuhiro Yoshimura, Yasunori Enomoto, Hideki Yasui, Hironao Hozumi, Masato Karayama, Yuzo Suzuki, Kazuki Furuhashi, Noriyuki Enomoto, Yutaro Nakamura, Naoki Inui, Hiromitsu Sumikawa, Takeshi Johkoh, Takafumi Suda

**Affiliations:** 1 Second Division, Department of Internal Medicine, Hamamatsu University School of Medicine, Hamamatsu, Japan; 2 Department of Clinical Pharmacology and Therapeutics, Hamamatsu University School of Medicine, Hamamatsu, Japan; 3 Department of Radiology, Sakai City Medical Center, Sakai, Japan; 4 Department of Radiology, Kansai Rosai Hospital, Amagasaki, Japan; Toranomon Hospital, JAPAN

## Abstract

**Background:**

Spontaneous pneumothorax is a complication that occurs in patients with connective tissue disease-associated interstitial lung disease (CTD-ILD); however, few studies on the clinical implications of pneumothorax for patients with CTD-ILD have been performed.

**Objectives:**

This study aimed to investigate the incidence and prognostic significance of pneumothorax and the risk factors for its onset in patients with CTD-ILD.

**Methods:**

This study included 140 consecutive patients with CTD-ILD. Clinical characteristics, laboratory findings, pulmonary function test results, and chest high-resolution computed tomography (HRCT) images were retrospectively evaluated.

**Results:**

A total of 18 patients (12.9%) developed pneumothorax during their clinical course. The cumulative incidence of pneumothorax from the time of CTD-ILD diagnosis was 6.5%, 8.7%, and 11.3% at 1, 3, and 5 years, respectively. The 10-year survival rate was significantly lower in patients with pneumothorax (29.6%) than that in those without pneumothorax (81.3%). The development of pneumothorax was significantly associated with poor prognosis (HR 22.0; p < 0.010). Furthermore, a lower body mass index, greater extent of reticular abnormalities on HRCT, and administration of methylprednisolone pulse therapy were significantly associated with the development of pneumothorax.

**Conclusion:**

Pneumothorax is a serious complication in the clinical course of patients with CTD-ILD and the onset of pneumothorax predicts a poor outcome.

## Introduction

Interstitial lung disease (ILD) is one of the common complications in patients with connective tissue disease (CTD), such as rheumatoid arthritis (RA), polymyositis (PM)/dermatomyositis (DM), Sjögren’s syndrome (SjS), and systemic scleroderma (SSc) [1]. ILD is associated with impaired respiratory function and leads to an increased risk of mortality in various CTDs [2]; thus, it is clinically important to determine the factors associated with poor outcomes in patients with CTD-associated ILD (CTD-ILD). Several studies have demonstrated the prognostic significance of clinical data, including older age, male sex, and lower level of forced vital capacity (FVC), in patients with CTD-ILD [3–7].

Pneumothorax sometimes occurs in patients with CTD-ILD during their clinical course. Generally, secondary spontaneous pneumothorax induces respiratory failure more frequently than primary spontaneous pneumothorax [8–12]. Furthermore, the recurrence rate of secondary spontaneous pneumothorax is considered to be higher than that of primary spontaneous pneumothorax [10, 12]. We have previously reported that pneumothorax developed in 20.2% of patients with idiopathic pulmonary fibrosis (IPF) during their clinical courses, and the onset of pneumothorax was significantly associated with a poor outcome [13]. The onset of pneumothorax may also affect the clinical course and prognosis of patients with CTD-ILD; however, there have been no studies on the incidence and clinical importance of pneumothorax in patients with CTD-ILD. In addition, few studies have assessed the risk factors for the onset of pneumothorax in patients with CTD-ILD. In general, the male sex, a tall and thin stature, a history of smoking, and the presence of subpleural bullae are considered to be risk factors of primary spontaneous pneumothorax [8, 12, 14]. However, whether these factors are associated with the onset of pneumothorax in patients with CTD-ILD remains uncertain, as pre-existing diseases and drugs used for treatment may affect the conditions. In the present study, we investigated the incidence and prognostic significance of pneumothorax in patients with CTD-ILD. In addition, risk factors for the onset of pneumothorax were assessed in patients with CTD-ILD.

## Methods

### Study subjects

Clinical records were retrospectively reviewed for 140 consecutive patients with CTD-ILD, who were evaluated at Hamamatsu University Hospital (Hamamatsu, Japan) between January 2000 and December 2014. All data were fully anonymized before we accessed them. Medical records were accessed between May 2018 and May 2019. The diagnoses of CTDs were based on accepted criteria[15–18], according to clinical findings. For RA or SjS, we have reassessed according to the criteria [15, 18] and confirmed all the patients with RA or SjS in this study were fulfilled the criteria. The diagnosis of ILD was based on the presence of respiratory symptoms, physical examination findings, chest high-resolution computed tomography (HRCT) findings, and pulmonary function tests. Other diseases, such as infection, drug-induced pneumonia, aspiration-induced lung injury, and heart failure, were excluded. No patients had concurrent advanced cancer at the time of CTD-ILD diagnosis. This study was conducted in accordance with the ethical standards of the Declaration of Helsinki and approved by the Institutional Review Board of Hamamatsu University School of Medicine (approval no. 15–197). The Institutional Review Board waived patient approval or informed consent as the study involved a retrospective review of patient records.

### Clinical data collection

We retrospectively reviewed the patients’ medical records, laboratory data, pulmonary function data, and lung HRCT image findings obtained at the time of ILD diagnosis. We also reviewed treatments for CTD-ILD, such as the use of prednisolone and immunosuppressive agents (e.g., cyclophosphamide, cyclosporin, tacrolimus, azathioprine, and methotrexate), during the observation period.

### Radiological analysis

HRCT images were obtained from all the patients on initial diagnosis of ILD and were reviewed by two expert thoracic radiologists with 30- and 15-years, respectively, who had no access to the patients’ clinical data. The slice thickness of the HRCT was 1.0 or 1.5-mm and the section interval was 10-mm. The HRCT findings, including the presence of honeycombing, reticular pattern, emphysema, ground-glass opacity (GGO), and consolidation, were interpreted according to the Fleischner Society criteria [19]. The extent of reticular abnormalities (i.e., reticular pattern and honeycombing), emphysema, GGO, and consolidation were semi-quantitatively scored as follows: Grade 0 (0%), Grade 1 (<25%), Grade 2 (25–50%), Grade 3 (50–75%), and Grade 4 (>75%). During the observation period, the patients underwent chest radiography or HRCT at least every 6 months. The clinical records and all radiological images (chest radiographs or HRCT images) were reviewed by three pulmonologists to confirm the onset of pneumothorax.

### Statistical analysis

Data are presented as the number (percentage) or median (interquartile ranges). Either Fisher’s exact test or the Mann-Whitney U test was used for between-group comparisons. The cumulative incidence of pneumothorax was evaluated using Gray’s test, considering any cause of death as an important competing factor. The observation period for survival was calculated from the date of initial ILD diagnosis to the final date of contact or time of death. Univariate and multivariate Cox proportional hazards model analyses were performed to determine the predictive factors affecting the prognosis and onset of pneumothorax. The onset of pneumothorax, use of prednisolone, immunosuppressive agents and methylprednisolone pulse therapy were analyzed as time-dependent covariates. The Kaplan–Meier method was used to produce survival curves, and the survival rates between the groups of patients were compared with a log-rank test. A p-value of <0.05 was considered statistically significant for all analyses. All statistical analyses were performed with EZR (Saitama Medical Centre, Jichi Medical University, Saitama, Japan), which is a graphical user interface for R software (version 2.13.0, The R Foundation for Statistical Computing, Vienna, Austria) [20].

## Results

### Clinical characteristics

The baseline characteristics of the study subjects at the time of CTD-ILD diagnosis are shown in Table 1. The median age was 62.5 years, and 61 patients (43.6%) were male. The median observation period was 72.2 months. During their clinical course, 18 patients (12.9%) developed pneumothorax and 122 patients did not. There were no significant differences in baseline characteristics between the patients with pneumothorax and those without pneumothorax, although body mass index (BMI) and Krebs von den Lungen–6 (KL-6) levels tended to be lower in the patients with pneumothorax. The underlying CTDs among the 140 patients comprised RA in 44 patients, PM/DM in 48 patients, SjS in 16 patients, SSc in 15 patients, and overlap syndrome in 17 patients. There were no significant differences in the incidence of pneumothorax among the underlying diseases (6.8% in RA, 10.4% in PM/DM, 18.8 in SjS, 20.0% in SSc, and 23.5% in overlap syndrome; S1 Fig).

**Table 1:** Baseline characteristics of the study subjects.

	Total	Pneumothorax (+)	Pneumothorax (-)	
	n = 140	n = 18	n = 122	*P*-value
Age, years	62.5 (56–71)	57.5 (54–64)	63 (56–71)	0.229
Male, n (%)	61 (43.6)	8 (44.4)	53 (43.4)	1.000
BMI, kg/m^2^	22.3 (20.2–24.6)	21.1 (18.2–24.1)	22.3 (20.3–24.8)	0.112
Pulmonary function tests				
FVC, %predicted	76.1 (62.9–88.3)	69.1 (60.4–78.7)	77 (64.0–89.0)	0.182
FEV_1_, %predicted	76.9 (64.75–86.73)	70.8 (63.0–85.1)	78 (65.5–86.7)	0.518
Laboratory tests				
KL-6, U/ml	806 (522–1279)	544 (401–1180)	821 (531–1303)	0.100
PaO_2_ on room air, mmHg	76.8 (68.4–85)	80.1 (69.0–82.0)	76.55 (67.9–85.0)	0.898
BAL findings				
Neutrophils, %	1.0 (0.3–3.3)	1.0 (0.5–5.8)	1.0 (0.2–3.0)	0.400
Lymphocytes, %	6.2 (3.8–11.3)	5.1 (4.1–8.1)	6.3 (3.8–12.6)	0.437
Eosinophils, %	0.9 (0.3–2.8)	0.7 (0.2–1.0)	1.0 (0.4–3.3)	0.195
HRCT findings				
Extent of GGO, 0/1/2/3/4	21/93/15/8/3	3/12/0/2/1	18/81/15/6/2	0.208
Extent of consolidation, 0/1/2/3/4	77/56/7/0/0	11/7/0/0/0	66/49/7/0/0	0.767
Extent of emphysema, 0/1/2/3/4	98/35/6/0/0	15/2/1/0/0	83/33/5/0/0	0.788
Extent of reticular abnormalities, 0/1/2/3/4	64/46/27/3/0	7/5/6/0/0	57/41/21/3/0	0.334
CTD diagnosis, RA/PMDM/SjS/SSc/overlap	44/48/16/15/17	3/5/3/3/4	41/43/13/12/13	0.214
Treatment				
Prednisolone, n (%)	110 (78.6)	14 (77.8)	96 (78.7)	1.000
Methylprednisolone pulse therapy, n (%)	52 (37.1)	10 (55.6)	42 (34.4)	0.116
Immunosuppressive agents, n (%)	79 (56.4)	11 (61.1)	68 (55.7)	0.801
Observation period, months	72.2 (48.3–116.9)	34.5 (3.2–70.9)	81.8 (50.5–118.6)	0.012

Data are presented as the median (interquartile ranges) or number (%).

BAL, bronchoalveolar lavage; BMI, body mass index; CTD, connective tissue disease; FEV_1_, forced expiratory volume in 1 s; FVC, forced vital capacity; GGO, ground-glass opacity; HRCT, high-resolution computed tomography; KL-6, Krebs von den Lungen–6; PaO_2_, partial pressure of oxygen in arterial blood; PMDM, polymyositis/dermatomyositis; RA, rheumatoid arthritis; SjS, Sjögren’s syndrome; SSc, systemic scleroderma.

### Treatments and outcomes

Approximately 80% of the patients received prednisolone, and >50% of the patients received both prednisolone and immunosuppressive agents (Table 1). Fifty-two patients (37.1%) were administered with intravenous methylprednisolone pulse therapy (1 g/day for 3 days). The treatment regimens did not differ significantly between the two groups. A total of 33 patients (12 with pneumothorax and 21 without pneumothorax) died during the observation period. The mortality rate was significantly higher in patients with pneumothorax than in those without (66.7% vs. 17.2%, respectively; p < 0.001). The mortality rate was similar among the underlying diseases (20.5% for RA, 20.8% for PM/DM, 31.3% for SjS, 26.7% for SSc, and 29.4% for overlap (S1 Table). In all CTDs, the mortality rate was higher in patients with pneumothorax than in those without pneumothorax (S1 Table).

### Cumulative incidence of pneumothorax in CTD-ILD

**Fig 1:**
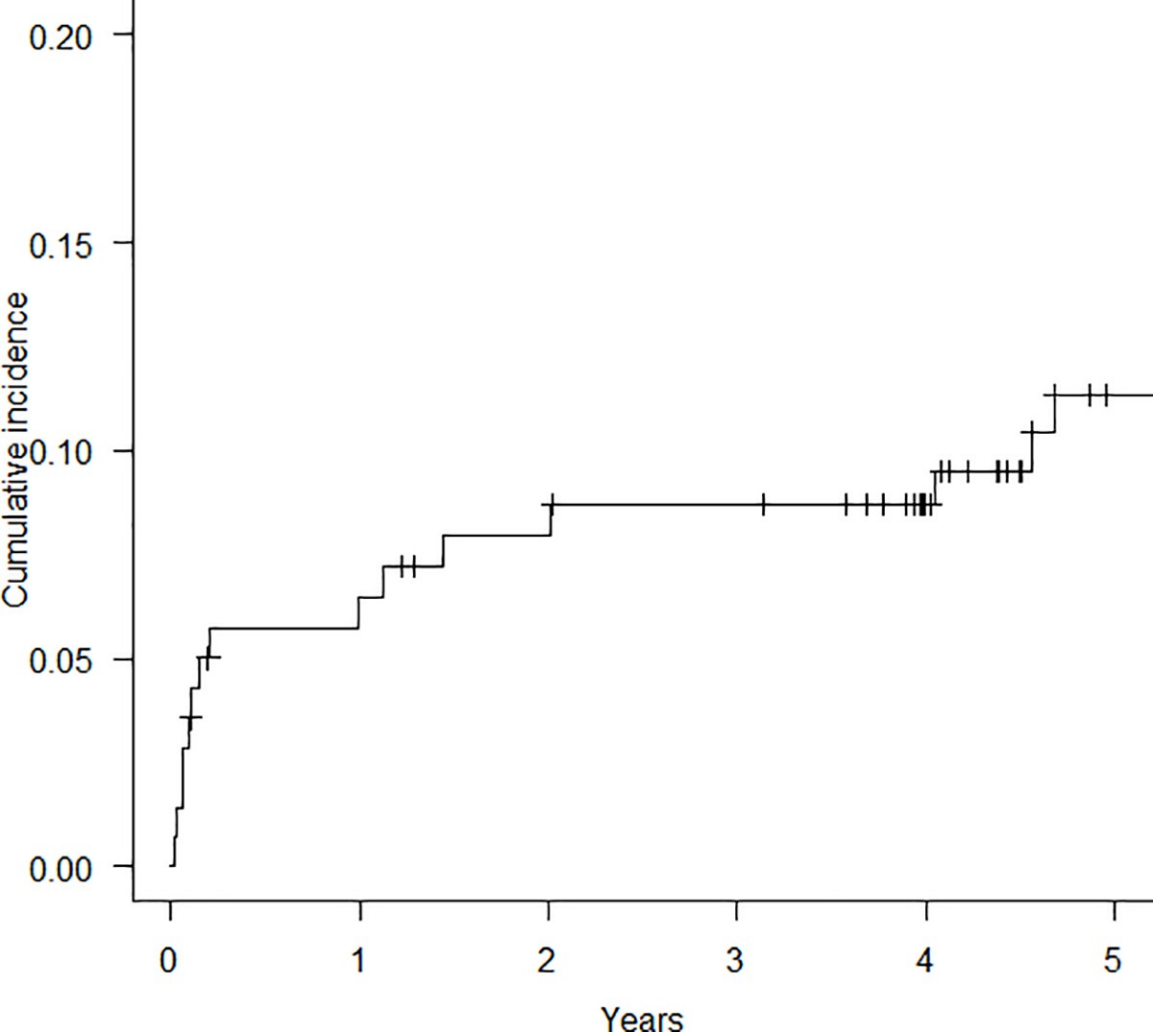
Cumulative incidence of pneumothorax from the time of connective tissue disease-associated interstitial lung disease diagnosis. The cumulative incidence of pneumothorax was 6.5%, 8.7%, and 11.3% at 1, 3, and 5 years from diagnosis, respectively.

The cumulative incidence of pneumothorax from the time of CTD-ILD diagnosis was 6.5%, 8.7%, and 11.3% at 1, 3, and 5 years, respectively (Fig 1). In the patients with pneumothorax, nine patients (50%) developed pneumothorax within 1 year of CTD-ILD diagnosis.

### Prognostic significance of pneumothorax in CTD-ILD

The results of the univariate Cox proportional hazards models are shown in Table 2. Male sex, lower %FVC levels, lower PaO_2_ levels, and the extent of reticular abnormalities (≥Grade 2) were significantly associated with a poor outcome in patients with CTD-ILD (Table 2). Notably, pneumothorax was strongly associated with a poor prognosis (HR 22.0; 95%CI 10.3–46.9; p < 0.001). Furthermore, multivariate analyses, following adjustment for these factors, revealed that the onset of pneumothorax was independently associated with a poor prognosis in the patients with CTD-ILD (Table 3).

**Table 2:** Univariate analyses for the overall survival of patients with CTD-ILD.

	HR	95%CI	*P*-value
Age, per 1 year increase	1.03	0.99–1.06	0.160
Male	2.04	1.02–4.07	0.044
BMI, per 1 kg/m^2^ increase	0.92	0.82–1.04	0.175
Pulmonary function test			
FVC (% predicted), per 1% increase	0.97	0.95–0.99	0.016
FEV_1_ (% predicted), per 1% increase	0.98	0.95–1.00	0.054
Laboratory tests			
KL-6, per 10 U/mL increase	1.00	0.99–1.01	0.243
PaO_2_ on room air, per-10 mm Hg increase	0.95	0.93–0.98	<0.001
BAL findings			
Neutrophils, per 1% increase	1.04	0.98–1.10	0.202
Lymphocytes, per 1% increase	0.99	0.96–1.03	0.747
Eosinophils, per 1% increase	0.96	0.83–1.11	0.567
HRCT findings			
Extent of GGO, Grade ≥2	1.53	0.66–3.54	0.320
Extent of consolidation, Grade ≥2	2.58	0.90–7.39	0.079
Extent of emphysema, Grade ≥2	1.09	0.26–4.62	0.909
Extent of reticular abnormalities, Grade ≥2	2.48	1.20–5.14	0.015
CTD diagnosis			
Rheumatoid arthritis	0.85	0.40–1.84	0.684
Polymyositis/Dermatomyositis	0.77	0.36–1.62	0.486
Sjögren's syndrome	1.20	0.46–3.13	0.707
Systemic scleroderma	1.31	0.46–3.77	0.615
Pneumothorax	22.0	10.3–46.9	<0.001

BAL, bronchoalveolar lavage; BMI, body mass index; CI, confidence interval; CTD-ILD, connective tissue disease-associated interstitial lung disease; FEV_1_, forced expiratory volume in 1 s; FVC, forced vital capacity; GGO, ground-glass opacity; HR, hazard ratio; HRCT, high-resolution computed tomography; KL-6, Krebs von den Lungen-6; PaO_2_, partial pressure of oxygen in arterial blood.

**Table 3:** Multivariate models for predictors of mortality in patients with connective tissue disease-associated interstitial lung disease.

		Model 1			Model 2			Model 3	
	HR	95%CI	*P*-value	HR	95%CI	*P*-value	HR	95%CI	*P*-value
Age, per 1 year increase	1.01	0.96–1.05	0.872	1.02	0.97–1.06	0.527	1.02	0.98–1.06	0.298
Male	2.45	1.05–5.72	0.038	2.16	0.97–4.83	0.059	1.88	0.91–3.87	0.087
FVC (% predicted), per 1% increase	0.98	0.95–1.00	0.063						
PaO_2_ on room air, per 10 mm Hg increase				0.94	0.91–0.96	<0.001			
Extent of reticular abnormalities, Grade ≥2							2.12	1.01–4.44	0.046
Pneumothorax	16.8	6.92–40.9	<0.001	32.6	13.0–81.7	<0.001	21.6	10.0–46.4	<0.001

CI, confidence interval; FVC, forced vital capacity; HR, hazard ratio; PaO_2_, partial pressure of oxygen in arterial blood.

**Fig 2:**
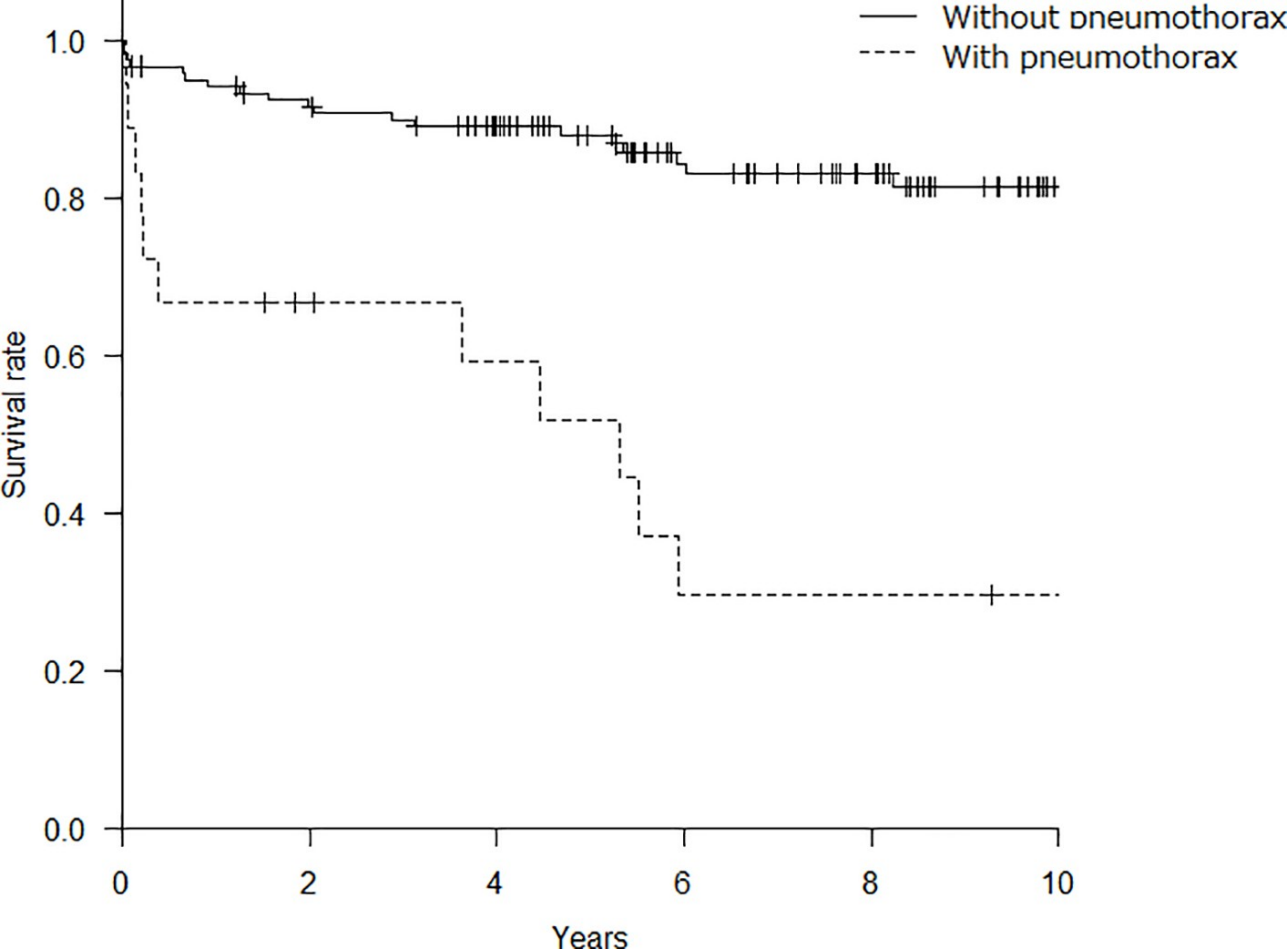
Kaplan-Meier survival curve in the patients with pneumothorax and those without pneumothorax. The 5-year and 10-year survival rate was significantly lower in patients with pneumothorax than those without pneumothorax (5-year: 51.9% vs. 88.0%, 10-year: 29.6% vs. 81.3%, p < 0.001).

Comparison of the survival curves between the patients with pneumothorax and those without pneumothorax are shown in Fig 2. The 5-year and 10-year survival rates from the ILD diagnosis were significantly lower in patients with pneumothorax than those in patients without pneumothorax (5-year: 51.9% vs. 88.0%, 10-year: 29.6% vs. 81.3%, p < 0.001). The median survival time from the onset of pneumothorax in patients with pneumothorax was 15.2 months (S2 Fig). In this study, pneumothorax developed 21 times (in 18 patients) including recurrence. Eight cases (38%) were improved by the rest, and 6 (29%) were by thoracic drainage. The median drainage period was 14.5 days (interquartile ranges 8.5–16) in 6 cases who were improved only by thoracic drainage. Seven (33%) of the 21 cases were refractory to thoracic drainage. Among them, pleurodesis (using minocycline, beriplast, autologous blood) were performed in 2 cases, however, they could not get reexpansion of the lungs after pleurodesis. Subsequently, they received surgical operation. One patient improved, however the other died after surgical operation. Five of 7 patients who were refractory to thoracic drainage could not be performed pleurodesis and surgery because of their poor general condition, and required chest drainage up to their time of death.

### Risk factors for the onset of pneumothorax in CTD-ILD

Cox proportional hazards model analysis was performed to determine the factors that contribute to the onset of pneumothorax in patients with CTD-ILD (Table 4). Methylprednisolone pulse therapy was significantly associated with the onset of pneumothorax (HR 3.40; 95%CI 0.85–5.96; p = 0.010). In 14 patients who developed pneumothorax during corticosteroid therapy (including methylpredonizolone pulse therapy), median period between start of corticosteroid therapy and onset of pneumothorax was 34 days (interquartile ranges 14–398 days). A lower BMI and greater extent of reticular abnormalities (≥Grade 2) tended to be associated with the onset of pneumothorax (HR 0.86 and 2.41; 95% CI 0.73–1.02 and 0.90–6.49; p = 0.083 and 0.081, respectively). The multivariate analyses demonstrated that methylprednisolone pulse therapy, lower BMI, and greater extent of reticular abnormalities (≥Grade 2) were independent predictors of the onset of pneumothorax in patients with CTD-ILD (S2 Table).

**Table 4:** Univariate analyses of the predictive factors for the onset of pneumothorax in patients with CTD-ILD.

	HR	95%CI	*P*-value
Age, per 1 year increase	0.99	0.95–1.03	0.662
Male	1.20	0.47–3.04	0.705
BMI, per 1 kg/m^2^ increase	0.86	0.73–1.02	0.083
Pulmonary function tests			
FVC (% predicted), per 1% increase	0.98	0.95–1.01	0.106
FEV_1_ (% predicted), per 1% increase	0.98	0.95–1.01	0.277
Laboratory tests			
KL-6, per 10 U/mL increase	0.99	0.99–1.01	0.337
PaO_2_ on room air, per 10 mmHg increase	0.98	0.94–1.02	0.318
BAL findings			
Neutrophils, per 1% increase	1.01	0.92–1.11	0.790
Lymphocytes, per 1% increase	0.94	0.84–1.05	0.258
Eosinophils, per 1% increase	0.75	0.47–1.21	0.225
HRCT findings			
Extent of GGO, Grade ≥2	1.19	0.34–4.11	0.786
Extent of consolidation, Grade ≥2	0.65	0.28–1.54	0.330
Extent of emphysema, Grade ≥2	1.20	0.16–9.04	0.860
Extent of reticular abnormalities, Grade ≥2	2.41	0.90–6.49	0.081
CTD diagnosis			
Rheumatoid arthritis	0.46	0.13–1.58	0.215
Polymyositis/Dermatomyositis	0.67	0.24–1.89	0.448
Sjögren's syndrome	1.44	0.41–5.04	0.564
Systemic scleroderma	1.74	0.50–6.10	0.385
Treatment			
Prednisolone	2.47	0.78–7.83	0.126
Methylprednisolone pulse therapy	3.40	1.32–8.74	0.011
Immunosuppressive agents	2.24	0.85–5.96	0.105

BAL, bronchoalveolar lavage; BMI, body mass index; CI, confidence interval; CTD-ILD, connective tissue disease-associated interstitial lung disease; FEV_1_, forced expiratory volume in 1 s; FVC, forced vital capacity; GGO, ground-glass opacity; HR, hazard ratio; HRCT, high-resolution computed tomography; KL-6, Krebs von den Lungen-6; PaO_2_, partial pressure of oxygen in arterial blood.

## Discussion

In the present study, we examined the incidence, prognostic significance of pneumothorax and risk factors for the onset of pneumothorax in a cohort of 140 consecutive patients with CTD-ILD. Our findings revealed that pneumothorax was a common complication (cumulative incidence of pneumothorax: 6.5%, 8.7%, and 11.3% at 1, 3, and 5 years, respectively) and onset of pneumothorax was a significant prognostic factor in patients with CTD-ILD. In addition, it was found that a lower BMI, greater extent of reticular abnormalities on HRCT and administration of methylprednisolone pulse therapy were risk factors for the onset of pneumothorax. This study highlights the clinical importance of pneumothorax as a serious complication associated with poor outcomes in patients with CTD-ILD, of which clinicians should be aware in the clinical course of CTD-ILD.

To date, there have been few studies demonstrating the clinical implications of pneumothorax in patients with CTD-ILD. The present study revealed for the first time, to the best of our knowledge, that the onset of pneumothorax was strongly associated with poor prognosis in patients with CTD-ILD (HR 22.0; Table 2). In the cohort of 140 patients with CTD-ILD, the 5-year survival rate was 83.4%, which was consistent with the result of a former study involving 93 patients with CTD-ILD (28 with RA, 35 with SSc, eight with PM/DM, 11 with SjS, one with systemic lupus erythematosus, five with mixed connective tissue disease, and five with “other”) [21]. However, the 5-year survival rate of patients with pneumothorax in the present study was only 51.9%, which was significantly lower than that of the patients without pneumothorax (88.0%). In addition, our data demonstrated that the survival time from the onset of pneumothorax was relatively short (median survival time: 15.2 months), which was almost identical for the survival time from the onset of pneumothorax in patients with IPF in our previous study (median survival time: 13.3 months). Therefore, clinicians should pay attention to the onset of pneumothorax in the clinical course of patients with CTD-ILD, as its onset highly influences patient prognosis.

At present, the incidence of pneumothorax in patients with CTD-ILD remains unclear. The present study demonstrated that the cumulative incidence of pneumothorax from the time of CTD-ILD diagnosis was 6.5%, 8.7%, and 11.3% at 1, 3, and 5 years, respectively. Previous studies have reported the incidence of pneumothorax in patients with IPF as 6.4–34% [13, 22, 23]. We have previously reported that the cumulative incidence of pneumothorax in patients with IPF was 8.5% and 17.7% at 1 and 3 years, respectively. Based on the findings of former studies and the present study, although the incidence of pneumothorax in CTD-ILD is likely to be marginally lower than that in IPF, pneumothorax is a common and serious complication in patients with CTD-ILD.

It was important to elucidate the predictive factors for the onset of pneumothorax as our findings demonstrated that pneumothorax was significantly associated with poorer outcomes in patients with CTD-ILD. In the present study, we found that a lower BMI was associated with the onset of pneumothorax in patients with CTD-ILD, which is consistent with previous reports regarding primary spontaneous pneumothorax [8, 12, 14] and secondary pneumothorax in patients with IPF [13]. A lower BMI is considered to be a risk factor for the development of blebs, one of the causes of pneumothorax [24]. In addition, an association was found between reticular abnormalities on HRCT and pneumothorax in the patients with CTD-ILD. These findings are also consistent with the previous reports regarding secondary pneumothorax in patients with IPF [13, 23].

The present study also showed that methylprednisolone pulse therapy was associated with the onset of pneumothorax in patients with CTD-ILD. In the real-world, corticosteroid therapy, including methylprednisolone pulse therapy, is often required for patients with CTD-ILD in their clinical courses, although corticosteroid may increase a risk of pneumothorax. In this viewpoint, clinician should pay more attention to the onset of pneumothorax in patients with CTD-ILD, especially those who have risk factors for the development of pneumothorax in their clinical findings (e.g. a lower body mass index, greater extent of reticular abnormalities on HRCT, and administration of methylprednisolone pulse therapy). In general, the etiological factors of primary spontaneous pneumothorax, such as emphysema-like changes and pleural porosity, are known to contribute to air leakage into the pleural space [12, 25–28]. Inflammatory processes accelerate these etiologies, suggesting that a persistent inflammatory process in the lung causes the development of pneumothorax in CTD-ILD. As patients administered methylprednisolone pulse therapy usually have higher disease activity, pneumothorax may be more likely in these patients. In addition, corticosteroids impair the healing process of pneumothorax by decreasing vascular permeability and suppressing the migration of inflammatory cells [29]. Therefore, corticosteroid use is likely to be associated with refractory pneumothorax; however, the association between corticosteroid use and the onset of pneumothorax remains unclear. Further investigation is required to determine whether methylprednisolone pulse therapy itself affects the onset of pneumothorax or disease activity.

In this cohort, 33% of the cases with pneumothorax in CTD-ILD were refractory to thoracic drainage, and most of them could not be performed pleurodesis and/or surgery because of their poor general condition. Picado *et al*. reported that it was not easy to get reexpansion of the lungs for patients with pneumothorax in ILD even though thoracic drainage were performed [30], which is consistent with the findings in the present study. Alternative therapeutic approach should be considered for patients with pneumothorax refractory to thoracic drainage. Recently, the efficacy of Endobronchial Watanabe Spigot (EWS) has been reported for patients with refractory pneumothorax in ILD [31]. The combination of such treatments might be effective for patients with refractory pneumothorax in CTD-ILD, although further studies will be required.

There were limitations to this study. First, the study was retrospective, and the observation period differed among patients, which may affect the incidence of pneumothorax. Although this bias exists, the median observation period of the study subjects was 72.2 months (Table 1), which was considered long enough to evaluate the incidence of pneumothorax in their clinical course. Second, the sample size of each CTD was relatively small, therefore, it was not possible to perform detailed analysis based on CTD diagnosis. However, the results revealed no obvious difference in the incidence of pneumothorax among CTDs (Table 1; S1 Fig), and the patients with pneumothorax tended to have a poorer prognosis than those without pneumothorax for each CTD (S1 Table). In addition, none of the CTDs were associated with the onset of pneumothorax in patients with CTD-ILD (Table 4). Taken together, the results of this study revealed that pneumothorax was a complication indicative of a poor prognosis for any type of CTD-ILD. Larger prospective observational studies are required to validate these findings.

In conclusion, pneumothorax is a serious complication in the clinical course of CTD-ILD, which is strongly associated with a poor survival rate. The incidence of pneumothorax in patients with CTD-ILD was relatively high, and clinicians should pay attention to the onset of pneumothorax, particularly in the patients who have risk factors for its onset.

## Supporting information

S1 Fig Incidence rate of pneumothorax for each patient with CTD. (TIF)

S2 Fig Kaplan-Meier survival curve from the onset of pneumothorax in patients with pneumothorax. The median survival time was 15.2 months.
(TIFF)

S1 Table Mortality rate for each patient with CTD. (DOCX)

S2 Table Multivariate analysis of the predictive factors for the onset of pneumothorax in patients with CTD-ILD. (DOCX)
