# Supplementary figures and images for: Pneumothorax in connective tissue disease-associated interstitial lung disease
Source: PLoS One. 2020 Jul 7;15(7):e0235624. doi: 10.1371/journal.pone.0235624 (PMC7340294; doi:10.1371/journal.pone.0235624)

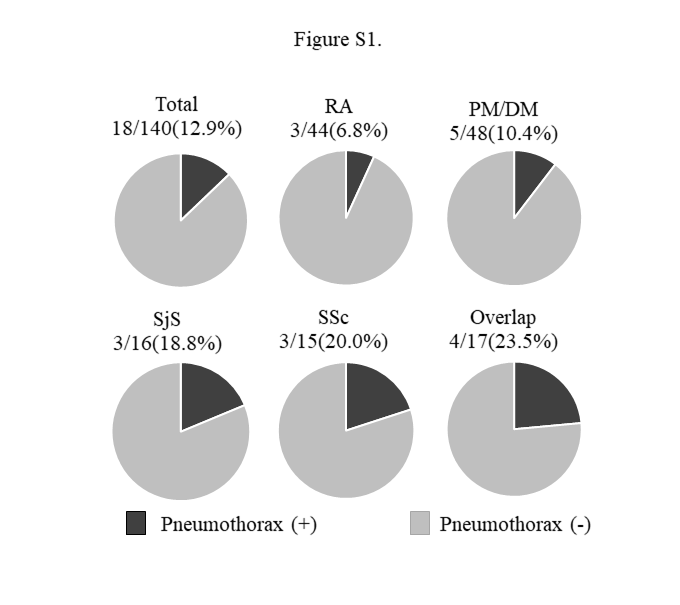

Supplement: S1 Fig — (TIF) [file pone.0235624.s001.tif]

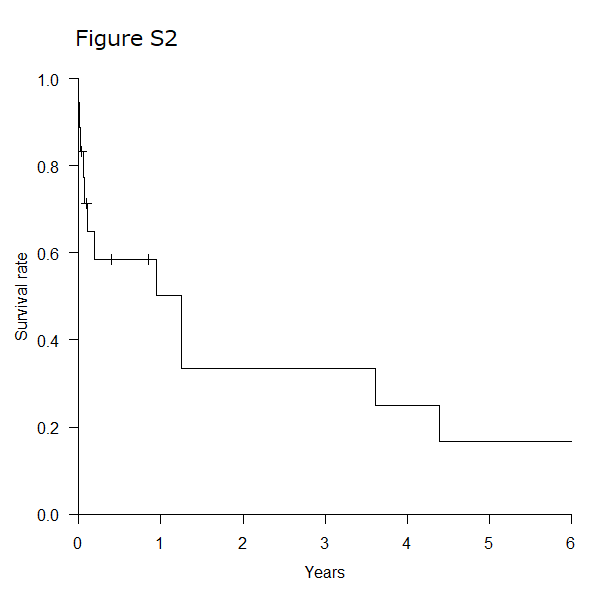

Supplement: S2 Fig — The median survival time was 15.2 months. (TIFF) [file pone.0235624.s002.tiff]
