# Supplementary material for: Pneumothorax in connective tissue disease-associated interstitial lung disease
Source: PLoS One. 2020 Jul 7;15(7):e0235624. doi: 10.1371/journal.pone.0235624 (PMC7340294; doi:10.1371/journal.pone.0235624)
Supplement: S1 Table — (DOCX) [file pone.0235624.s003.docx]

**S1 Table.**  Mortality rate for each patient with CTD.

|  | Total | Pneumothorax(+) | Pneumothorax(-) |
| --- | --- | --- | --- |
| Rheumatoid arthritis | 9/44(20.5) | 2/3(66.7) | 7/41(17.1) |
| Polymyositis/Dermatomyositis | 10/48(20.8) | 4/5(80.0) | 6/43(14.0) |
| Sjögren's syndrome | 5/16(31.3) | 2/3(66.7) | 3/13(23.1) |
| Systemic scleroderma | 4/15(26.7) | 2/3(66.7) | 2/12(16.7) |
| Overlap | 5/17(29.4) | 2/4(50.0) | 3/13(23.1) |
