# Supplementary material for: Pneumothorax in connective tissue disease-associated interstitial lung disease
Source: PLoS One. 2020 Jul 7;15(7):e0235624. doi: 10.1371/journal.pone.0235624 (PMC7340294; doi:10.1371/journal.pone.0235624)
Supplement: S2 Table — (DOCX) [file pone.0235624.s004.docx]

**S2 Table.** Multivariate analysis of the predictive factors for the onset of pneumothorax in patients with CTD-ILD.

|  | HR | 95%CI | p-value |
| --- | --- | --- | --- |
| BMI, per 1-kg/m^2^ increase | 0.85 | 0.73-0.99 | 0.046 |
| Reticular score ≥2 | 3.11 | 1.11-8.71 | 0.031 |
| Methylprednisolone pulse therapy | 4.72 | 1.74-12.8 | 0.002 |

BMI, body mass index; CI, confidence interval; FVC, forced vital capacity; HR, hazard ratio.
